# Contribution of the Cerebellum and the Basal Ganglia to Language Production: Speech, Word Fluency, and Sentence Construction—Evidence from Pathology

**DOI:** 10.1007/s12311-020-01207-6

**Published:** 2020-10-29

**Authors:** Maria Caterina Silveri

**Affiliations:** Department of Psychology, Università Cattolica del Sacro Cuore, Largo Agostino Gemelli 1, 20123 Milano, Italy

**Keywords:** Cerebellum, Basal ganglia, Speech, Word fluency, Agrammatism, Network pathology

## Abstract

Evidence reported in recent decades increasingly confirms that both the cerebellum and the basal ganglia, which are primarily involved in movement control, also have a significant role in a vast range of cognitive and affective functions. Evidence from pathology indicates that the disorders of some aspects of language production which follow damage of the cerebellum or respectively basal ganglia, i.e., disorders of speech, word fluency, and sentence construction, have identifiable neuropsychological profiles and that most manifestations can be specifically attributed to the dysfunctions of mechanisms supported by one or the other of these structures. The cerebellum and the basal ganglia are reciprocally interconnected. Thus, it is plausible that some disorders observed when damage involves one of these structures could be remote effects of abnormal activity in the other. However, in a purely clinical-neuropsychological perspective, primary and remote effects in the network are difficult to disentangle. Functional neuroimaging and non-invasive brain stimulation techniques likely represent the indispensable support for achieving this goal.

## Introduction

The aim of this short review is to explore whether some disorders of language production associated with cerebellum and basal ganglia damage, respectively, present, under a behavioral point of view, identifiable profiles and whether they can be reinterpreted in the context of a “dysfunctional network.”

After I make a few historical references, I will consider the current view that attributes a significant role to the cerebellum and basal ganglia not only in motor control but also in the modulation of cognitive functions. First, I will briefly describe the neural base of motor and non-motor control. Then, I will mention the new neurofunctional perspective, i.e., that the cerebellum and basal ganglia are components of a highly integrated network. In this perspective, I will consider the principal neuropsychological disorders of language production, i.e., disorders of speech, word fluency, and sentence construction, in cerebellar and basal ganglia damage (see Table 1). With a few necessary exceptions, a discussion of neuroimaging and non-invasive brain stimulation studies is beyond the scope of this review.

**Table 1:** The main reports on speech, word fluency, and sentence construction disorders associated to cerebellar and basal ganglia damage, as described by the authors; reference in square brackets

Author(s)	Language disorder	Brain structures involved/type of pathology/syndrome
	Speech	
Darley et al., 1969 [1]	Ataxic dysarthria	Cerebellar disorders
	Hypokinetic dysarthria	Parkinsonism
Holmes et al., 2000 [2]	Hypokinetic dysarthria	Parkinson’s disease (PD)
Benke et al., 2000 [3]	Repetitive speech phenomena	PD
Perez-Lloret et al., 2012 [4]	Dysarthria associated to non-speech motor deficit	PD
Critchley EM, 1981 [5]	Dysarthria associated to non-speech motor deficit	Parkinsonism
Ackermann et al., 2014 [6]	Hypokinetic dysarthria, non-speech motor deficit, vocal and non-vocal aspects of emotional aspects during speech	Basal ganglia
van Lancker Sidtis et al., 2006 [7]	Dysprosody	Vascular lesion of basal ganglia
Casper et al., 2007 [8]	Dysprosody	Cerebellar ataxia
Skodda et al., 2009 [9]	Dysprosody	PD
Ciabarra et al., 2000 [10] Tany and Sakay, 2010 [11]	Stuttering	Subcortical and cerebellar vascular lesion
Juste et al., 2018 [12]	Stuttering	PD
Toft and Dietrichs, 2001 [13]	Stuttering	Subthalamic deep brain stimulation in PD
Yang et al., 2016 [14]	Stuttering	Developmental (cerebellum-basal ganglia thalamo cortical network)
Marien et al., 2018 (review) [15]	Neurogenic “Foreign accent syndrome”(FAS)	Vascular lesion of left motor/premotor cortex, basal ganglia and cerebellum
Keulen et al., (2017) (review and new cases) [16]	Neurogenic FAS	Posterior fossa damage
Priftis et al., 2020 [17]	Pure FAS	Right cortico-subcortical (lenticular) vascular lesion and diaschisis in the right thalamus and left cerebellum
Riva and Giorgi, 2000 [18]	Mutism	Surgery for cerebellar vermis medulloblastoma
Riva, 1998 [19]	Mutism	Cerebellitis
	Word fluency	
Leggio et al., 2000 [20]	Reduced phonemic word fluency	Vascular or degenerative damage of cerebellum
Neau et al., 2000 [21]	Reduce word fluency (letter)	Vascular damage of cerebellum (unilateral left/right/bilateral)
Schweizer et al., 2010 [22]	Reduced word fluency	Vascular damage (right cerebellum)
Peterburs et al., 2010 [23]	Reduced word fluency(letter)	Vascular damage (cerebellar unilateral left/unilateral right/bilateral)
Azuma et al., 1997 [24]	Reduced letter fluency	PD
Auriacombe et al., 1993 [25]	Reduced category fluency	PD
McDow et al., 2011 [26] Pettit et al., 2013 [27]	No direct comparison between letter and semantic fluency	PD
Obeso et al., 2012 [28]	No difference between phonemic and category word fluency	PD
Henry JD, Crawford, 2004 (meta-analysis) [29]	Semantic fluency more impaired than letter fluency	PD
Ho et al., 2001 [30]	Reduced verbal fluency (decreased phonemic switching)	Huntington disease (HD)
Radanovic and Mansur, 2017 [31]	Reduced verbal fluency (in aphasic patients)	Vascular lesion of basal ganglia
	Sentence construction	
Silveri et al., 1994 [32] Marien et al., 199 [33] Zettin et al., 1997 [34] Gasparini et al., 1999 [35]	Agrammatism	Vascular lesion (right cerebellum)
Justus et al., 2004 [36]	Subclinical deficit in grammatical morphology	Vascular and degenerative cerebellar damage
Troche et al., 2012 [37]	Sentence simplification	PD
Murray and Lenz, 2001 [38]	Sentence simplification	PD and HD
Giavazzi et al., 2018 [39]	Altered selection of grammatical morphemes	HD
Hinzen et al., 2018 [40]	Sentence simplification	HD
Dick et al., 2018 [41]	No syntactic impairment	PD
	Verb deficit	
Signorini et al., 2006 [42] Silveri et al., 2012 [43] Cousin et al., 2018 [44] Garcia et al., 2018 [45] Crescentini et al., 2008 [46] Colman et al., 2009 [47]	Deficit in processing the word class “verbs”	PD
Cotelli et al., 2007 [48]	Deficit in processing the word class “verbs”	Parkinsonism
Frank et al., 2007 [49]	Preservation of the processing of the word class “verbs”	Acute cerebellar lesion
Richter et al., 2004 [50]	Preservation of the processing of the word class “verbs”	Cerebellar atrophy

## Cerebellum and Basal Ganglia in Movement and Cognition

Since the earliest investigations of the cerebellum, this brain structure has been considered responsible for the control of movement [51, 52]. Disturbances of accuracy and coordination are the hallmark of cerebellar movement disorders [53, 54], which are key elements of the ataxic syndrome. The first detailed and systematic descriptions of patients with various manifestations of the cerebellar syndrome were made by Holmes who, working as a physician in France during the World War I (reported in [55]) (for a review, see [56]), described many patients with cerebellar injuries due to gunshot wounds. Although from that time on the relationship between lesion site and the respective symptomatology was taken for granted in the case of the cerebellum, to some extent in analogy to what is now known as the brain-lesion methodology [57], things turned out differently for other structures involved in movement control, i.e., the basal ganglia. Probably due to the objective difficulty of identifying and localizing a neurodegenerative process at that time, damage to the substantia nigra was associated with Parkinson’s disease (PD) symptomatology [58] more than a century after the seminal observation by James Parkinson [59]. Although inferring the function of a brain structure by the symptom its lesion generates might be misleading, we can at least assume that the cerebellum and basal ganglia have different roles in movement control given the different syndromes their respective lesions can produce.

With regard to the cerebellum, over time complementary and sometimes conflicting interpretations have been proposed for the complex motor syndrome of the cerebellar patient, with key concepts focused on disorders of timing, sensory and motor acquisition, and sensorimotor synchronization (for a consensus paper, see [54]). The cerebellum and the cerebral cortex are connected by feed-forward cerebellum-thalamus-cortico-ponto-cerebellar pathways. Despite the uniform cytoarchitecture and general principles of organization [60], segregated functional zones emerge in the complexity of the cerebellar connections to heterogeneous extracerebellar structures [61]. An influential hypothesis, which represents a formalization of the concept of prediction of future states, assumes that through mechanisms of learning the cerebellum acquires “internal models” of movement that, in turn, can automatically implement programming in the motor cortex; in other words, the cerebellum compares the actual with the predicted movement it has learned through sensory feedbacks [62, 63]. The hypothesis assumes that the implementation of internal models also extends to the cognitive domains, including language [64–67].

The connections between the topographically organized cerebellar cortex and the association areas primarily in the frontal and parietal regions represent the neural substrates of the cerebellar non-motor functions. The cognitive deficit that emerges following cerebellar lesions is largely driven by the type of cognitive ability which is specifically supported by the cortical areas the damaged cerebellar region is connected to and which the cerebellum modulates by monitoring timing and synchronization [68, 69] in order to ensure the adherence of the outcome to predictions. In the presence of a cerebellar lesion, the mismatch between prediction and performance is the basis for a “dysmetric” behavior [64], i.e., a core feature of the cerebellar cognitive impairment.

With regard to the basal ganglia, classical models assume that the control of movement initiation in the motor cortex is modulated by the balance of the inhibitory and facilitatory effects of the striopallidal networks on the thalamus [70, 71]. However, the role of the basal ganglia is not only in the initiation but also in the selection of actions by the suppression of the competing ones [72] (for review, see [73]). Multiple neuronal loops connect the dorsolateral prefrontal cortex to the head of the caudate nucleus (dorsal striatum) and then come back to the dorsolateral prefrontal cortex via the internal pallidal segment, substantia nigra pars reticulate, and thalamus [74]. Through these connections, the basal ganglia could participate in the selection of the appropriate motor program. In the cognitive domain, damage to the basal ganglia is expected to upset the complex balance between activation and inhibition that is at the base of any selection process aimed at goal-directed behaviors [75], which is the characterizing feature of the so-called executive function (see also [76]).

### Cerebellum and Basal Ganglia: a Highly Integrated Functional Network

For many years, studies of the cerebellum and basal ganglia were carried out in parallel, primarily in patients with cerebellar or extrapyramidal syndromes. Only in relatively recent times has the complex neurofunctional interaction of these structures started to emerge, primarily in neuroanatomical studies of non-human primates. These studies suggest that their respective functions could be reframed within a basal-ganglia-cerebellar-cortical network [77–79]. Both the cerebellum and the basal ganglia project by means of distinct thalamic nuclei to the same primary motor areas with somatotopical organization; however, most outputs from the basal ganglia and the cerebellum are directed to areas other than the motor areas [80], i.e., the premotor, temporal, and parietal cortex [81, 82]. These multisynaptic systems of connections are organized according to a closed-loop architecture [77] with reciprocal communication between the neocortical areas, cerebellum, and basal ganglia [83]; however, the cerebellar-cortical and the basal ganglia-cortical circuitry should maintain their independence [84]. The cerebellum and basal ganglia not only interact by means of reciprocal connections with the cerebral cortex, they are also densely interconnected at the subcortical level: the dentate nucleus has multisynaptic connections with the striatum and the globus pallidus through the thalamus [85] and the subthalamic nucleus projects to the cerebellar cortex through the pontine nuclei [77] (see also [79] for a review on the role of the thalamus in the cerebellum-basal ganglia interaction and [86] for a consensus paper on the interplay between the cerebellum, basal ganglia, and cerebral cortex).

In a functional network, pathological changes at one site can produce remote effects [78]. In fact, damage to the cerebellum is considered responsible for some motor and non-motor manifestations in PD [87] and dystonia [88]; and parkinsonian symptoms, on the other hand, could be part of cerebellar ataxia [89]. Moreover, functional studies of asymptomatic carriers with the SCA2 mutation show increased cortico-cerebellar connectivity, which has been interpreted as a compensatory mechanism in the early stages of a dopamine deficit [90, 91]. Overall, these studies confirm that the cerebellum and basal ganglia are components of a highly integrated cortical-subcortical functional network that supports movement control, and, on the basis of an expanding literature, they suggest that this network might also subtend cognitive and affective behavior [83, 92–100]. Nevertheless, the dynamic interplay between these two structures and their relationship to the cerebral cortex are not getting the attention they deserve [86], particularly in cognitive domains.

## Speech Disorders

### Ataxic and Hypokinetic Dysarthria

The speech disorder of a cerebellar patient has been termed *ataxic dysarthria* [1]. According to Ziegler et al. [101], the structuralist position that considers the phoneme as an “immaterial entity” has largely neglected the sensory (auditory) and motor aspects of speech [102]. Indeed, a neurobiological vision of phonology enhances the physical dimension of phonemes and their relation to speech movements [103]. A dysregulation of sensorimotor control, which is the principal feature of the cerebellar motor syndrome, has been considered the core of ataxic dysarthria [1]. It is mainly traced back to altered articulation and phonation, resulting in hesitant, scanned, laborious, and explosive speech [104]. In fact, the cerebellar role in motor control also affects vocal tract movements [105]. The original hypothesis is that the cerebellum intervenes in processing the proprioceptive feedback necessary for monitoring the movements of the oral tract during speech production, facilitating their adherence to the motor patterns acquired and stabilized in the motor cortex by the frequency of their occurrence in the speaker’s language [106]. Resolving the mismatch between expected and actual movement should drive the learning process aimed at improving performance [60]. The contribution of the cerebellum can be reframed within the “internal model hypothesis,” extending its role in sensory motor control to the speech domain [65]; in other words, the cerebellum learns the internal model and automatically implements the programs of the motor cortex during speech production. The cerebellum also processes time speech parameters [107, 108] and discriminates speech sounds that are crucial for processing phonological stimuli [109].

Most descriptions of speech disorders due to basal ganglia damage refer to PD. Monotonous loudness and pitch, imprecise articulation, hypophonia and dysphonia, and short rushes of speech constitute the main features of parkinsonian *hypokinetic dysarthria* [1, 2]. Repetitive speech phenomena have also been reported in later stages [3]. The frequent association of dysarthria with non-speech motor deficits of the orobuccal apparatus, such as dysphagia and sialorrhea [4]), suggests that hypokinetic dysarthria is only one of the symptoms of a broader disorder that is not limited to the vocal tract but involves articulation, phonation, breathing, and swallowing [5], as well as vocal and non-vocal aspects (facial, gestural) of emotional expression during speech production [6] (see also [110] for the contribution of the cortico-ponto-cerebellar system). The elaboration of timing parameters in both motor and perception is not only a competence of the cerebellum but also of the basal ganglia [111–113]; however, there is no evidence of a specific role of the basal ganglia in monitoring time parameters in the language domain.

Ataxic and hypokinetic dysarthria should both be distinguished from apraxia of speech, a disorder of speech planning [101]. Respiratory and phonatory disorders are part of the symptom set of both ataxic dysarthria and hypokinetic dysarthria but are negligible in apraxia of speech [114, 115]), thus confirming the pure “cortical” nature of the latter. However, despite some peculiarities and the different localization of lesions producing ataxic dysarthria, hypokinetic dysarthria, and apraxia of speech, respectively (right vermal and paravermal lesions vs. striatum vs. left insula, motor, premotor, and supplementary motor cortex) [116–120]), apraxia of speech, ataxic dysarthria, and hypokinetic dysarthria share some semiological characteristics [121, 122]. Furthermore, impaired sensorimotor integration, which has been indicated as the principal feature of ataxic dysarthria, may also be a component of parkinsonian hypokinetic dysarthria [123]. As suggested by Ziegler (in [105]), similarities among disorders of speech following lesions in different brain structures might reflect universal aspects of motor impairment or compensatory mechanisms. However, the dense cortical and subcortical interconnections between the cerebellum and basal ganglia make alternative interpratations plausible: speech articulation is supported by a functional network in which the cerebellum, basal ganglia, and left anterior frontal cortex participate in a highly integrated way; some symptoms can be attributed to specific competences of the cerebellum, basal ganglia, or cerebral cortex, respectively; others cannot be easily attributed to any of these structures, and an interpretation based on a dysfunctional network seems more appropriate.

### Dysprosody, Stuttering, and the “Foreign Accent Syndrome”

Prosody, the melody of speech [7], is a speech dimension primarily related to duration parameters in the production of syllables, syllabic stress, and pitch (fundamental frequencies). *Dysprosody* due to an alteration of duration parameters (acceleration and slowing) of syllable production, speech intensity, and pitch variation has been reported in cerebellar ataxia [8]; dysprosody associated with hypophonia is also a common feature of speech production in PD [9, 124] and is also frequently reported in vascular lesions encompassing the basal ganglia (see [7] for a review and description of two cases).

However, on the basis of the available literature, it is not easy to identify differences in the prosodic disorders of patients with cerebellar and basal ganglia damage, respectively. The literature indicates that cortical structures might also be involved in the control of prosody, suggesting that this aspect of production is underpinned by an extensive functional cortico-subcortical network. This is also true of another disorder of speech, i.e., acquired *stuttering*, which is the involuntary repetition of sounds and speech blocks while speaking. It can appear as a consequence of cortical (premotor) subcortical [10] and right cerebellar lesions of vascular nature [11] and in neurodegenerative disease such as PD [12] primarily after a deep (subthalamic) brain simulation (ST-DBS) [13]. Large involvement of the cerebellar and basal ganglia-thalamocortical network has also been demonstrated in developmental forms of stuttering [14].

The neurogenic *foreign accent syndrome* (FAS) is the most typical disorder of prosody; in this case, following a brain lesion, the subject starts to speak with an accent that is perceived as “foreign” by speakers of the same language community. It should be considered a syndrome in its own right, i.e., independent of, although frequently associated with, dysarthria, apraxia of speech, or aphasia [125]. This syndrome is principally observed in vascular lesions encompassing the left premotor and motor cortex and/or the basal ganglia but also the parietal and temporal cortex (see [15] for a review) and cerebellum (see [16] for a review and new cases). Occasionally, lesions have been described in the right hemisphere (Critchley, 1962—reported in [16, 17]). In a recent FAS [17] report, a right cortico-subcortical (lenticular nucleus) vascular lesion was associated with diaschisis in the right thalamus and left cerebellum.

Overall, these observations suggest that a cortico-subcortical neural network which includes both the cerebellum and basal ganglia and the thalamus and cerebellar-cortical and cortico-striato-pallidal thalamic connections might participate in the monitoring of planning, coordination, timing, sequencing, and selection of the appropriate motor programs during the implementation of these relatively peripheral stages of verbal production. Patterns of combinations of primary lesions and remote effects might generate these different expressions of speech disorders that could be among those best interpreted as network deficits. To the right hemisphere might be attributed a role in the emotional-affective components of prosody [126], which are also supported by a cortico-ponto-cerebellar network [110].

### Cerebellar Mutism

The *cerebellar mutism* syndrome is a profound speech disorder that has been observed after the resection of large midline tumors (medulloblastoma) in children [18, 127] (see also [128] for review) or cerebellitis [19]. It can be associated with oropharyngeal dyspraxia and ataxia and various manifestations of the so-called cognitive affective syndrome of the cerebellar patient [93]. It is usually transient, but residual dysarthria may be a permanent symptom. Transient ischemia encompassing the dento-thalamo-cortical pathways or cerebellar deep nuclei due to vasospasm or cytotoxic edema is a possible cause. Involvement of the basal ganglia in cerebellar mutism is suggested by the sudden resolution of the syndrome when midazolam is administered; this is supposed to produce indirect cortical excitation by inhibiting the thalamocortical pathway via the striatum [129].

## Verbal Fluency

*Verbal fluency* is primarily a verbal task [130]. In the classical version, it requires generating unique words within a time limit according to a given criterion that can be a semantic category (*category or semantic fluency*) or a letter of the alphabet (*letter or phonemic fluency*). Different patterns of performance are observed in individuals with different brain pathologies and localization, which suggests that category and phonemic fluency reflect the status of different underlying cognitive systems. Category fluency is largely based on an automatic search in the lexical semantic system that might help organize a list of semantic items, e.g., a shopping list, in an ecological context [131]. Letter fluency, by contrast, requires greater attention because it requires an active lexical search based on the given phoneme without any contextual associative support [132]. Clinically, category fluency is considered an indicator of lexical-semantic integrity, whereas letter fluency is currently used as a measure of executive abilities in which the active lexical search requires keeping and monitoring information in the working memory system.

Leggio et al. [20] demonstrated that word fluency is reduced in the presence of cerebellar damage when the criterion is a letter of the alphabet, but not when the criterion is a semantic category. To explain this pattern, the authors emphasized the role of the cerebellum in the acquisition of novel strategies for processing sequenced information, such as that required to organize the lexical search, without any facilitation of automatic searching routines, as in semantic fluency. During the lexical search, the cerebellum processes a large amount of information from the cerebral cortex (probably supported by a specific subcomponent of working memory [133]), in order to identify time and sequence coincidences that would allow obtaining the correct phonemic cluster [134]. The acquired time and sequence coincidence might represent the internal model by which the cerebellum optimizes the performance. The involvement of the cerebellum in phrasal prediction (i.e., the probability that a word will recall a second word on the basis of temporal contiguity and predictability of words in discourse) is also consistent with the disproportionate impairment of letter compared to semantic fluency [67].

The decline of letter compared to category fluency in cerebellar patients is quite a robust finding [21–23]. The pattern observed in patients with basal ganglia damage, primarily PD patients, seems less consistent. In the context of a general reduction of word fluency, some studies report reduced letter compared to category fluency [24]; others report the opposite pattern [25]; and in others, letter and semantic fluency are not directly compared [26, 27] or no differences emerge [28]. However, a meta-analysis conducted on a large number of studies of PD subjects found a disproportionate impairment of category compared to letter fluency, interpreted as a semantic memory disorder [29], due to difficulties in accessing stored semantic information [135]. A dysfunctional frontostriatal pathway is considered the base of the dysexecutive disorder in PD patients [136], whereas cognitive control of semantic access has its neural substrate in the connection between the inferior frontal gyrus (IFG) and the temporal lobe [137, 138]. This pathway modulates lexical access and selection processes primarily in the left hemisphere [139, 140]. In particular, the uncinate fasciculus, i.e., the connection between the IFG and the temporal lobe, is damaged in PD [141]. Thus, a dysregulation of the interaction between the frontostriatal-frontotemporal circuitry, which reduces the inhibition of competing alternatives [142], might be consistent with PD patients’ difficulty in selecting items from lexical semantic memory. In conclusion, the basal ganglia should provide some contextual facilitation from semantic memory during word production through the interplay of the frontostriatal and frontotemporal pathways. A dysfunctional circuitry could weaken the inhibition of competing alternatives within the semantic domains during the lexical/semantic search [143], indirectly increasing the strength of the interference of the competing alternatives and ultimately reducing the efficiency of word selection.

Reduced verbal fluency has also been documented in patients with other types of parkinsonism such as Huntington Disease (HD) [30]. In this pathology, reduced verbal fluency has been attributed to decreased phonemic switching, whereas semantic switching and both phonemic and semantic clustering remain stable over time; this pattern has been attributed to damage of the frontostriatal network.

Reduced word fluency has also been described in vascular lesions of the basal ganglia [31] but in the context of an aphasic disorder (see also [144, 145] for a discussion). This makes difficult any comparison with patients with cerebellar lesions or neurodegenerative pathology of the basal ganglia, which, in spite of language disorders, cannot be considered aphasic patients. Moreover, no direct comparison between letter and semantic fluency was carried out systematically in these studies.

Finally, both the cerebellum and the basal ganglia support the lexical search process even though they seem to have different roles. However, the available evidence is to some extent inconsistent and indefinitive. Therefore, caution should be taken in drawing conclusions.

## Sentence Construction

### Agrammatism

*Agrammatism*, sometimes identified with Broca’s aphasia, is a language disorder that is characterized by reduced speech fluency with effortful speech output, sentence construction simplification, and morphosyntactic errors. According to Miceli [146], two orders of hypotheses have been put forward to interpret this disorder: loss of linguistic knowledge (primarily syntactic and morphological) or, alternatively, reduction of the cognitive resources needed to elaborate this knowledge.

Although this approach is not without criticism [146], these two orders of hypotheses can help explain the agrammatic syndromes described in patients with cerebellar lesions and, to some extent, the syntactic difficulties that have sometimes been noted in patients with basal ganglia damage, such as in PD. Independent evidence from the declarative/procedural model of language [147] assumes that mental grammar involves procedural learning and is built up by rule-governed combinations of words in sequential and hierarchical combinations. According to this model, implementation of mental grammar is supported by networks that include the left frontal cortex, basal ganglia, and cerebellum; however, no assumption is made about the specific roles of each of these structures, primarily the basal ganglia and the cerebellum.

A few patients have been described with agrammatic speech following cerebellar damage [32–35] (see also [148] for review); subclinical agrammatic disorders have been also documented [36]. Although they do not completely overlap, the single cases described (and to some extent also those with subclinical syndromes) shared some clinical characteristics, such as lesion localization in the right cerebellar hemisphere and the “anterior” nature of the language deficit, with reduced fluency, dysarthria, and agrammatism with prevalent morphological disorders and word-finding difficulties. The case reported by Marien et al. [33] also presented characteristics of dynamic aphasia, with a dissociation between spontaneous and imposed language. All patients recovered completely within a few months, with the exception of Marien et al.’s patient, who, although improved, still had some residual difficulties one year later. A working memory disorder has been identified as the main cause of the agrammatic production in cerebellar patients, with impaired timing between selection of grammatical morphemes and application of syntactic rules [32, 34, 35]); a similar explanation is that of a disorder at a high level of articulatory planning, as a reinterpretation of the “economy of the effort” hypothesis, which would generate a delay in the application of syntactic rules [34]. A different interpretation was proposed by Marien et al. [33]. These authors hypothesized a “deactivation” of the linguistic function in the left prefrontal cortex due to loss of excitatory pathways from the cerebellum; thus, they assigned a direct role in the functional organization of language to the cerebellum. The agrammatic syndrome of cerebellar patients could also be tentatively reframed within the “internal model” hypothesis. The cerebellum encodes the syntactic-grammatical rules governing the production of connected speech in its syntactic and morphological aspects and continuously monitors the sentences planned in the left frontal cortex-Broca’s area. The cerebellum’s failure to monitor due to deterioration of the predicted schema of the sentence and errors signal could underlie the emergence of the uncorrected sentence.

Damage to the basal ganglia in PD impairs language “without a linguistic deficit per se” (pg. 914) [149], and this is consistent with the reduced sentence complexity described in this pathology [38, 37] without evidence of specific syntactic limitation [41], thus resizing the hypothesis of a direct role of the basal ganglia in sentence construction. Similarly, observations from HD do not indicate disorders of sentence construction consistent with the loss of grammatical knowledge but rather reduced accuracy in selecting grammatical alternatives, with more frequent selection of suboptimal alternatives than healthy controls [39] or a simplification of sentence production [40]. Although different patterns in sentence construction might be expected on the basis of the different pathological circuitry in PD and HD which gives rise to different syndromes, hypokinetic vs. hyperkinetic, no clear differences emerge in the direct comparison between PD and HD [38](however, see [147] for possible implications of altered verb morphology in sentence construction in PD and HD).

Coming back to the hypothesis of “loss of linguistic knowledge” vs. “reduction of cognitive resources,” the cerebellum and basal ganglia seem to respect this dichotomy. The cerebellum intervenes in sentence construction through segregated connections with Broca’s area; thus, damage to the cerebellum is followed by the dysfunction of Broca’s area and agrammatism; the dysfunction of the basal ganglia is associated with a general reduction of the cognitive control that results in sentence simplification or increased randomness in the selection of a grammatical alternative. More generally, the different influence of cerebellar and basal ganglia damage on sentence construction suggests that the cerebellum intervenes by means of dedicated connections with Broca’s area, whereas the basal ganglia guarantee general attentional support through the corticostriatal network. A dysfunctional Broca’s area will not allow for the planning of syntactically correct sentences, whereas dysfunctional corticostriatal pathways will result in sentence simplification as a non-specific manifestation of the dysexecutive syndrome; in fact, no evidence suggests the existence of a language-specific component of executive control.

### The Case of the Noun and Verb Grammatical Classes

Much literature indicates that the production of verbs is impaired compared to that of nouns in a series of brain pathologies (including parkinsonism) that involve the anterior regions of the brain because of frontostriatal dysfunction [42, 43, 48]. The hypothesis advanced (however, also see [44, 45]) is that verb processing is penalized not because of its specific lexical semantic nature, which requires the integrity of substrates involved in the motor representation of actions, but because of the intrinsically greater difficulty of verbs, which compared to nouns require the inhibition of a higher number of competitors in order to select the correct item [46]. Thus, in pathologies involving the basal ganglia, in which the cognitive disorder is dominated by the dysexecutive syndrome [150], verbs are penalized more than nouns [47]. In fact, in an experimental condition in which the production of nouns is more difficult than that of verbs, nouns are disproportionally impaired [142]. The disproportionate impairment of verbs is a consistent finding in extrapyramidal syndromes; the same cannot be said about cerebellar syndromes because there is a substantial lack of evidence of a specific deficit for this class of words [49, 50]. The cerebellum supports only specific components of the executive function, such as working memory (see [133] for a discussion), not the process of inhibition of irrelevant information, which is attributed to the corticostriatal network. This explains why the cerebellar lesion is not associated with the deficit for verb production.

## A Role for the Thalamus?

The abovementioned thalamic diaschisis observed in the foreign accent syndrome by Priftis et al. [17] suggests that the thalamus may have a role in language production disorders in patients with cerebellar or basal ganglia damage. The thalamus is the main subcortical interconnection between these two structures. Given the importance of the thalamus in language processing [151, 152], its role within the cerebellum-basal ganglia network should receive more attention. A set of thalamic nuclei is considered the hub that links the basal ganglia and cerebellum to the premotor and prefrontal cortex associated with language production (see [153] for a review). It is also well known that one of the most common negative effects of the ST-DBS for the treatment of severe motor symptoms in PD is the decline of word fluency (see [154] for a meta-analysis). Although the mechanism underlying this negative effect is still being debated, changes produced by ST-DBS in neuronal activity in the pallido-thalamic and cerebello-thalamic circuits [155] could be taken into account to better delineate the role of the thalamus in the linguistic deficits observed in the pathology of the cerebellum and basal ganglia.

## Conclusions

Evidence from pathology suggests that the neuropsychological profile of the various disorders of language production in cerebellar and basal ganglia damage, respectively, has mostly identifiable features. Although cerebellar and parkinsonian syndromes share some symptoms, most of them can be attributed to disorders supported by one or the other structure. Furthermore, even in the case of shared symptoms, it is not certain whether they can be interpreted as signs of a dysfunctional network or, instead, as general aspects of the disorder of language production (primarily when motor components are involved) or of the reduction of cognitive resources such as working memory. Functional neuroimaging may incorporate network effects [156] that could facilitate the identification of the remote effects of a lesion at a given site. Non-invasive techniques (such as TMS) have also been adopted to investigate the system-level interaction of the cerebellum and basal ganglia with some positive clinical implications for PD and dystonia (see [86] for a discussion). However, in a purely clinical and neuropsychological perspective, the effects of the primary lesion site cannot be easily disentangled from those in functionally connected regions.

There is no interpretative model in neuropsychology that simultaneously considers the role of the cerebellum and basal ganglia in cognition in an integrated network. Comparing patients with cerebellar or basal ganglia damage with patients affected by a degenerative pathology such as olivopontocerebellar/multisystem atrophy, in which the “system” involved is precisely the cerebellar-basal ganglia network [157], could provide some clues. However, functional neuroimaging is likely the indispensable support for achieving this goal. Similarly, the application of non-invasive transcranial neuromodulation techniques might help us to better understand the role of the cerebellum in language [158] and to explore “distance effects” of cerebellar stimulation on speech and language disorders attributed to basal ganglia damage, also in a clinical-rehabilitative perspective.

It should not be overlooked, as the most recent neuropsychological literature documents that for what concerns the cerebellum many reports describe patients with focal lesions and only in part with neurodegeneration; the opposite concerns the basal ganglia, where the observations derive primarily from patients with neurodegenerative pathology and to a lesser extent vascular etiology. This could potentially limit a direct comparison of the contribution of the cerebellum and basal ganglia to language production. It is also useful to note that the functional perspective of a highly integrated basal ganglia cerebellar network [78] is mostly supported by evidence from neurodegenerative and neuropsychiatric diseases, more suitable to be interpreted as network disorders than vascular damage.

Another potential limitation is the relatively little attention paid to the specific functions of the different regions of the cerebellum as well as the different nuclei of the basal ganglia. At least in neuropsychological studies, reference is usually made only to the hemispheric side of the damage, and with regard to the basal ganglia, reference is usually made to the striatum and its connections with the frontal cortex. However, the various aspects of verbal production are plausibly supported by specific structures of the cerebellum and basal ganglia, as well as by segregated circuitries. Also in this case, functional studies and more detailed descriptions of structural damage and connectivity are the necessary complement to the neuropsychological evidence in order to provide a clinical counterpart to neuroanatomy studies that propose the cerebellum and basal ganglia as an integrated functional system.

## References

[1] Darley FL, Aronson AE, Brown JR (1969). Differential diagnostic patterns of dysarthria. J Speech Hear Res.

[2] Holmes RJ, Oates JM, Phyland DJ, Hughes AJ (2000). Voice characteristics in the progression of Parkinson’s disease. Int J Lang Commun Disord.

[3] Benke T, Hohenstein C, Poewe W, Butterworth B. Repetitive speech phenomena in Parkinson's disease. J Neurol Neurosurg Psychiatry. 2000;69(3):319–24. 10.1136/jnnp.69.3.319. 10.1136/jnnp.69.3.319 PMC1737094 10945806

[4] Perez-Lloret S, Nègre-Pagès L, Ojero-Senard A, Damier P, Destée A, Tison F, Merello M, Rascol O, for the COPARK Study Group, COPARK study group (2012). Oro-buccal symptoms (dysphagia, dysarthria, and sialorrhea) in patients with Parkinson’s disease: preliminary analysis from the French COPARK cohort. Eur J Neurol.

[5] Critchley EM (1981). Speech disorders of parkinsonism: a review. J Neurol Neurosurg Psychiatry.

[6] Ackermann H, Hage SR, Ziegler W (2014). Brain mechanisms of acoustic communication in human and non human primates: an evolutionary perspective. Behav Brain Sci.

[7] Van Lancker Sidtis D, Pachana N, Cummings JL, Sidtis JJ (2006). Dysprosodic speech following basal ganglia insult: toward a conceptual framework for the study of the cerebral representation of prosody. Brain Lang.

[8] Casper MA, Raphael LJ, Harris KS, Geibel JM (2007). Speech prosody in cerebellar ataxia. Int J Lang Commun Disord.

[9] Skodda S, Rinsche H, Schlegel U (2009). Progression of dysprosody in Parkinson’s disease over time-a longitudinal study. Mov Disord.

[10] Ciabarra AM, Elkind MS, Roberts JK, Marshall RS (2000). Subcortical infarction resulting in acquired stuttering. J Neurol Neurosurg Psychiatry.

[11] Tani T, Sakai Y (2010). Stuttering after right cerebellar infarction: a case study. J Fluen Disord.

[12] Juste FS, Sassi FC, Costa JB, de Andrade CRF (2018). Frequency of speech disruptions in Parkinson’s disease and developmental stuttering: a comparison among speech tasks. PLoS One.

[13] Toft M, Dietrichs E (2011). Aggravated stuttering following subthalamic deep brain stimulation in Parkinson’s disease--two cases. BMC Neurol.

[14] Yang Y, Jia F, Siok WT, Tan LH (2016). Altered functional connectivity in persistent developmental stuttering. Sci Rep.

[15] Mariën P, Keulen S, Verhoeven J (2019). Neurological aspects of foreign accent syndrome in stroke patients. J Commun Disord.

[16] Keulen S, Mariën P, van Dun K, Bastiaanse R, Manto M, Verhoeven J (2017). The posterior fossa and foreign accent syndrome: report of two new cases and review of the literature. Cerebellum..

[17] Priftis K, Algeri L, Barachetti L, Magnani S, Gobbo M, De Pellegrin S (2020). Acquired neurogenic foreign accent syndrome after right-hemisphere lesion with left cerebellar diaschisis: a longitudinal study. Cortex..

[18] Riva D, Giorgi C (2000). The cerebellum contributes to higher functions during development: evidence from a series of children surgically treated for posterior fossa tumours. Brain.

[19] Riva D (1998). The cerebellar contribution to language and sequential functions: evidence from a child with cerebellitis. Cortex..

[20] Leggio MG, Silveri MC, Petrosini L, Molinari M (2000). Phonological grouping is specifically affected in cerebellar patients: a verbal fluency study. J Neurol Neurosurg Psychiatry.

[21] Neau JP, Arroyo-Anllo E, Bonnaud V, Ingrand P, Gil R (2000). Neuropsychological disturbances in cerebellar infarcts. Acta Neurol Scand.

[22] Schweizer TA, Alexander MP, Susan Gillingham BA, Cusimano M, Stuss DT (2010). Lateralized cerebellar contributions to word generation: a phonemic and semantic fluency study. Behav Neurol.

[23] Peterburs J, Bellebaum C, Koch B, Schwarz M, Daum I (2010). Working memory and verbal fluency deficits following cerebellar lesions: relation to interindividual differences in patient variables. Cerebellum..

[24] Azuma T, Bayles KA, Cruz RF, Tomoeda CK, Wood JA, McGeagh A, Montgomery EB (1997). Comparing the difficulty of letter, semantic, and name fluency tasks for normal elderly and patients with Parkinson’s disease. Neuropsychology..

[25] Auriacombe S, Grossman M, Carvell S, Gollomp S, Stern MB, Hurtig HI (1993). Verbal fluency deficits in Parkinson’s disease. Neuropsychology..

[26] McDowd J, Hoffman L, Rozek E, Lyons KE, Pahwa R, Burns J, Kemper S (2011). Understanding verbal fluency in healthy aging, Alzheimer’s disease, and Parkinson’s disease. Neuropsychology..

[27] Pettit L, McCarthy M, Davenport R, Abrahams S (2013). Heterogeneity of letter fluency impairment and executive dysfunction in Parkinson’s disease. Heterogeneity of letter fluency impairment and executive dysfunction in Parkinson’s disease. J Int Neuropsychol Soc.

[28] Obeso I, Casabona E, Bringas ML, Alvarez L, Jahanshahi M (2012). Semantic and phonemic verbal fluency in Parkinson’s disease: influence of clinical and demographic variables. Behav Neurol.

[29] Henry JD, Crawford JR (2004). Verbal fluency deficits in Parkinson’s disease: a meta-analysis. J Int Neuropsychol Soc.

[30] Ho AK, Sahakian BJ, Robbins TW, Barker RA, Rosser AE, Hodges JR (2002). Verbal fluency in Huntington’s disease: a longitudinal analysis of phonemic and semantic clustering and switching. Neuropsychologia..

[31] Radanovic M, Mansur LL (2017). Aphasia in vascular lesions of the basal ganglia: a comprehensive review. Brain Lang.

[32] Silveri MC, Leggio MG, Molinari M (1994). The cerebellum contributes to linguistic production: a case of agrammatic speech following a right cerebellar lesion. Neurology..

[33] Marien P, Saerens J, Nanhoe R, Moens E, Nagels G, Pickut BA (1996). Cerebellar induced aphasia: case report of cerebellar induced prefrontal aphasic language phenomena supported by SPECT findings. J Neurol Sci.

[34] Zettin M, Cappa SF, D'amico A, Rago R, Perino C, Perani D, Fazio F (1997). Agrammatic speech production after a right cerebellar haemorrhage. Neurocase..

[35] Gasparini M, Di Piero V, Ciccarelli O, Cacioppo MM, Pantano P, Lenzi GL (1999). Linguistic impairment after right cerebellar stroke: a case report. Eur J Neurol.

[36] Justus T (2004). The cerebellum and English grammatical morphology: evidence from production, comprehension, and grammaticality judgments. J Cogn Neurosci.

[37] Troche MS, Altmann LA. Sentence production in Parkinson’s disease: effects of conceptual and task complexity. Appl Psycholinguist. 2012;33:225–51.

[38] Murray LL, Lenz LP (2001). Productive syntax abilities in Huntington’s and Parkinson’s diseases. Brain Cogn.

[39] Giavazzi M, Daland R, Palminteri S, Peperkamp S, Brugières P, Jaquemot C (2018). The role of the striatum in linguistic selection: evidence from Huntington’s disease and computational modeling. Cortex..

[40] Hinzen W, Rosselló J, Morey C (2018). A systematic linguistic profile of spontaneous narrative speech in pre-symptomatic and early stage Huntington’s disease. Cortex..

[41] Dick J, Fredrick J, Man G, Huber EJ, Lee J (2018). Sentence production in Parkinson’s disease. Clin Linguist Phon.

[42] Signorini M, Volpato C (2006). Action fluency in Parkinson’s disease: a follow-up study. Mov Disord.

[43] Silveri MC, Ciccarelli N, Baldonero E, Piano C, Zinno M, Soleti F, Bentivoglio AR, Albanese A, Daniele A (2012). Effects of stimulation of the subthalamic nucleus on naming and reading nouns and verbs in Parkinson’s disease. Neuropsychologia..

[44] Cousins KAQ, Ash S, Grossman M (2018). Production of verbs related to body movement in amyotrophic lateral sclerosis (ALS) and Parkinson’s disease (PD). Cortex..

[45] García AM, Bocanegra Y, Herrera E, Moreno L, Carmona J, Baena A, Lopera F, Pineda D, Melloni M, Legaz A, Muñoz E, Sedeño L, Baez S, Ibáñez A (2018). Parkinson’s disease compromises the appraisal of action meanings evoked by naturalistic texts. Cortex..

[46] Crescentini C, Mondolo F, Biasutti E, Shallice T (2008). Supervisory and routine processes in noun and verb generation in nondemented patients with Parkinson’s disease. Neuropsychologia..

[47] Colman KS, Koerts J, van Beilen M, Leenders KL, Post WJ, Bastiaanse R (2009). The impact of executive functions on verb production in patients with Parkinson’s disease. Cortex..

[48] Cotelli M, Borroni B, Manenti R, Ginex V, Calabria M, Moro A (2007). Universal grammar in the frontotemporal dementia spectrum: evidence of a selective disorder in the corticobasal degeneration syndrome. Neuropsychologia.

[49] Frank B, Schoch B, Hein-Kropp C, Dimitrova A, Hövel M, Ziegler W (2007). Verb generation in children and adolescents with acute cerebellar lesions. Neuropsychologia..

[50] Richter S, Kaiser O, Hein-Kropp C, Dimitrova A, Gizewski E, Beck A, Aurich V, Ziegler W, Timmann D (2004). Preserved verb generation in patients with cerebellar atrophy. Neuropsychologia.

[51] Pearce JM (2009). Marie-Jean-Pierre Flourens (1794-1867) and cortical localization. Eur Neurol.

[52] Babinski J (1902). Sur le rôle du cervelet dans les actes volitionnels nécessitant une succession rapide de mouvements (diadococinésie). Rev Neurol (Paris).

[53] Manto M, Bower JM, Conforto AB, Delgado-García JM, da Guarda SN, Gerwig M (2012). Consensus paper: roles of the cerebellum in motor control--the diversity of ideas on cerebellar involvement in movement. Cerebellum..

[54] Bodranghien F, Bastian A, Casali C, Hallett M, Louis ED, Manto (2016). Consensus paper: revisiting the symptoms and signs of cerebellar syndrome. Cerebellum..

[55] Manto M, Haines D (2012). Cerebellar research: two centuries of discoveries. Cerebellum..

[56] van Gijn J. Symptomatology of cerebellar tumours; a study of forty cases. By Grainger Stewart T. (Registrar) and Gordon Holmes (Resident Medical Officer, National Hospital, Queen Square, London). Brain 1904; 27: 522-591. With the symptoms of acute cerebellar injuries due to gunshot injuries. By Gordon Holmes. Brain 1917; 40: 461.535. With the cerebellum of man. By Gordon Holmes. Brain 1939; 62:1-30. Brain. 2007; 130:4–7. 10.1093/brain/aw1345.

[57] Vaidya AR, Pujara MS, Petrides M, Murray EA, Fellows LK (2019). Lesion studies in contemporary neuroscience. Trends Cogn Sci.

[58] Hassler R (1938). Zur pathologie der paralysis agitans und des postencephalitschen Parkinsonismus. J Psychol Neurol.

[59] Parkinson J. An essay on the shaking palsy. 1817. In: Classical Article. J Neuropsychiatry Clin Neurosci. 2002;14(2):223–36. 10.1176/jnp.14.2.223 11983801

[60] Fiez JA (2016). The cerebellum and language: persistent themes and findings. Brain Lang.

[61] Grimaldi G, Manto M (2012). Topography of cerebellar deficits in humans. Cerebellum..

[62] Wolpert DM, Miall RC, Kawato M (1998). Internal models in the cerebellum. Trends Cogn Sci.

[63] Ito M (2008). Control of mental activities by internal models in the cerebellum. Nat Rev Neurosci.

[64] Hickok G (2012). Computational neuroanatomy of speech production. Nat Rev Neurosci.

[65] Argyropoulos GP (2016). The cerebellum, internal models and prediction in ‘non-motor’ aspects of language: a critical review. Brain Lang.

[66] Moberget T, Ivry RB. Cerebellar contributions to motor control and language comprehension: searching for common computational principles. Ann N Y Acad Sci. 2016;1369(1):154–71. 10.1111/nyas.13094. 10.1111/nyas.13094 PMC5260470 27206249

[67] Mariën P, Manto M (2018). Cerebellum as a master-piece for linguistic predictability. Cerebellum..

[68] Molinari M, Chiricozzi FR, Clausi S, Tedesco AM, De Lisa M, Leggio MG (2008). Cerebellum and detection of sequences, from perception to cognition. Cerebellum..

[69] Lupo M, Olivito G, Angelini L, Funghi G, Pignatelli F, Siciliano L, et al. Does the cerebellar sequential theory explain spoken language impairments? A literature review. Clin Linguist Phon. 2020:1–14. 10.1080/02699206.2020.1745285. 10.1080/02699206.2020.1745285 32290716

[70] Albin RL, Young AB, Penney JB (1989). The functional anatomy of basal ganglia disorders. Trends Neurosci.

[71] DeLong MR (1990). Primate models of movement disorders of basal ganglia origin. Trends Neurosci.

[72] Nambu A, Tachibana Y, Chiken S. Cause of parkinsonian symptoms: firing rate, firing pattern or dynamic activity changes? Basal Ganglia. 2015;5:1–6. 10.1016/j.baga.2014.11.001.

[73] McGregor MM, Nelson AB (2019). Circuit mechanisms of Parkinson’s disease. Neuron..

[74] Leisman G, Braun-Benjamin O, Melillo R (2014). Cognitive-motor interactions of the basal ganglia in development. Front Syst Neurosci.

[75] Delgado MR (2007). Reward-related responses in the human striatum. Ann N Y Acad Sci.

[76] Papagno C, Trojano L (2018). Cognitive and behavioral disorders in Parkinson’s disease: an update. I: cognitive impairments. Neurol Sci.

[77] Bostan AC, Dum RP, Strick PL (2013). Cerebellar networks with the cerebral cortex and basal ganglia. Trends Cogn Sci.

[78] Bostan AC, Strick PL (2018). The basal ganglia and the cerebellum: nodes in an integrated network. Nat Rev Neurosci.

[79] Hintzen A, Pelzer EA, Tittgemeyer M (2018). Thalamic interactions of cerebellum and basal ganglia. Brain Struct Funct.

[80] Hoover JE, Strick PL (1999). The organization of cerebellar and basal ganglia outputs to primary motor cortex as revealed by retrograde transneuronal transport of herpes simplex virus type 1. J Neurosci.

[81] Doya K (2000). Complementary roles of basal ganglia and cerebellum in learning and motor control. Curr Opin Neurobiol.

[82] Koziol LF, Budding D, Andreasen N, D'Arrigo S, Bulgheroni S, Imamizu H (2014). Consensus paper: the cerebellum’s role in movement and cognition. Cerebellum..

[83] Middleton FA, Strick PL (1994). Anatomical evidence for cerebellar and basal ganglia involvement in higher cognitive function. Science..

[84] Percheron G, François C, Talbi B, Yelnik J, Fénelon G (1996). The primate motor thalamus. Brain Res Brain Res Rev.

[85] Hoshi E, Tremblay L, Féger J, Carras PL, Strick PL (2005). The cerebellum communicates with the basal ganglia. Nat Neurosci.

[86] Caligiore D, Pezzulo G, Baldassarre G, Bostan AC, Strick PL, Doya K, Helmich RC, Dirkx M, Houk J, Jörntell H, Lago-Rodriguez A, Galea JM, Miall RC, Popa T, Kishore A, Verschure PFMJ, Zucca R, Herreros I (2017). Consensus paper: towards a systems-level view of cerebellar function: the interplay between cerebellum, basal ganglia, and cortex. Cerebellum..

[87] Wu T, Hallett M (2013). The cerebellum in Parkinson’s disease. Brain.

[88] Filip P, Lungu OV, Shaw DJ, Kasparek T, Bareš M. The mechanisms of movement control and time estimation in cervical dystonia patients. Neural Plast. 2013:908741. 10.1155/2013/908741. 10.1155/2013/908741 PMC3806519 24198973

[89] Pedroso JL, Braga-Neto P, de Souza PV, Barsottini OG (2013). The cerebellum in Parkinson’s disease and parkinsonism in cerebellar disorders. Brain..

[90] Wu T, Wang C, Wang J, Hallett M, Zang Y, Chan P (2013). Preclinical and clinical neural network changes in SCA2 parkinsonism. Parkinsonism Relat Disord.

[91] Mirdamadi JL (2016). Cerebellar role in Parkinson’s disease. J Neurophysiol.

[92] Fiez JA (1996). Cerebellar contributions to cognition. Neuron..

[93] Schmahmann JD, Sherman JC (1998). The cerebellar cognitive affective syndrome. Brain..

[94] Middleton FA, Strick PL (2000). Basal ganglia output and cognition: evidence from anatomical, behavioral, and clinical studies. Brain Cogn.

[95] Rapaport M, Reekum R, Mayberg H (2000). The role of the cerebellum in cognition and behavior: a selective review. J Neuropsychiatr Clin Neurosci.

[96] Mariën P, Baillieux H, De Smet HJ, Engelborghs S, Wilssens I, Paquier P (2009). Cognitive, linguistic and affective disturbances following a right superior cerebellar artery infarction: a case study. Cortex..

[97] Leiner HC (2010). Solving the mystery of the human cerebellum. Neuropsychol Rev.

[98] Arsalidou M, Duerden EG, Taylor MJ (2013). The centre of the brain: topographical model of motor, cognitive, affective, and somatosensory functions of the basal ganglia. Hum Brain Mapp.

[99] Leggio M, Olivito G, Manto M, Huisman TAGM (2018). Topography of the cerebellum in relation to social brain regions and emotions. The Cerebellum: From Embryology to Diagnostic Investigations. Handb Clin Neurol.

[100] Schmahmann JD (2019). The cerebellum and cognition. Neurosci Lett.

[101] Ziegler W, Aichert I, Staiger A (2012). Apraxia of speech: concepts and controversies. J Speech Lang Hear Res.

[102] Dell GS, Schwartz MF, Martin N, Saffran EM, Gagnon DA (1997). Lexical access in aphasic and nonaphasic speakers. Psychol Rev.

[103] Goldstein L, Byrd D, Saltzman E, Arbib MA (2006). The role of vocal tract gestural action units in understanding the evolution of phonology. Action to language via the mirror neuron system.

[104] Silveri MC. Speech deficit. In: Gruol DL, Koibuchi N, Manto M, Molinari M, Schmahmann JD, Shen Y, editors. Essentials of cerebellum and cerebellar disorders: a primer for graduate students. Berlin: Springer; 2016. pp. 477–80.

[105] Mariën P, Ackermann H, Adamaszek M, Barwood CH, Beaton A, Desmond J (2014). Consensus paper: language and the cerebellum: an ongoing enigma. Cerebellum..

[106] Mooshammer CC, Goldstein L, Nam H, McClure S, Saltzman E, Tiede M (2012). Bridging planning and execution: temporal planning of syllables. J Phon.

[107] Ivry RB, Keele WJ (1989). Timing functions of the cerebellum. Cogn Neurosci.

[108] Molinari M. Sequencing. In: Gruol DL, Koibuchi N, Manto M, Molinari M, Schmahmann JD, Shen Y, editors. Essentials of Cerebellum and Cerebellar Disorders: A Primer For Graduate Students. Berlin: Springer; 2016. pp. 397–402.

[109] Ackermann H, Mathiak K, Riecker A (2007). The contribution of the cerebellum to speech production and speech perception: clinical and functional imaging data. Cerebellum..

[110] Hasson U, Llano DA, Miceli G, Dick AS (2014). Does it talk the talk? On the role of basal ganglia in emotive speech processing. Behav Brain Sci.

[111] Cope TE, Grube M, Singh B, Burn DJ, Griffiths TD (2014). The basal ganglia in perceptual timing: timing performance in multiple system atrophy and Huntington’s disease. Neuropsychologia..

[112] Harrington DL, Rao MS. Timing in neurodegenerative disorders of the basal ganglia. In: Vatakis A, Allman MJ, editors. Time distortions in mind; temporal processing in clinical populations. Leiden: Brill; 2015. pp. 190–225.

[113] Paton JJ, Buonomano DV (2018). The neural basis of timing: distributed mechanisms for diverse functions. Neuron..

[114] Kent RD, Kent JF, Duffy JR, Thomas JE, Weismer G, Stuntebeck S (2000). Ataxic dysarthria. J Speech Lang Hear Res.

[115] Ogar J, Slama H, Dronkers N, Amici S, Gorno-Tempini ML (2005). Apraxia of speech: an overview. Neurocase..

[116] Ackermann H, Vogel M, Petersen D, Poremba M (1992). Speech deficits in ischaemic cerebellar lesions. J Neurol.

[117] Urban PP, Marx J, Hunsche S, Gawehn J, Vucurevic G, Wicht S, Massinger C, Stoeter P, Hopf HC (2003). Cerebellar speech representation: lesion topography in dysarthria as derived from cerebellar ischemia and functional magnetic resonance imaging. Arch Neurol.

[118] Josephs KA, Duffy JR, Strand EA, Whitwell JL, Layton KF, Parisi JE, Hauser MF, Witte RJ, Boeve BF, Knopman DS, Dickson DW, Jack CR Jr, Petersen RC (2006). Clinicopathological and imaging correlates of progressive aphasia and apraxia of speech. Brain..

[119] Josephs KA, Duffy JR, Strand EA, Machulda MM, Senjem ML, Master AV, Lowe VJ, Jack CR, Whitwell JL (2012). Characterizing a neurodegenerative syndrome: primary progressive apraxia of speech. Brain..

[120] Urban PP (2013). Speech motor deficits in cerebellar infarctions. Brain Lang.

[121] Mariën P, Beaton A (2014). The enigmatic linguistic cerebellum: clinical relevance and unanswered questions on nonmotor speech and language deficits in cerebellar disorders. Cerebellum Ataxias.

[122] Mariën P, Verhoeven J (2007). Cerebellar involvement in motor speech planning: some further evidence from foreign accent syndrome. Folia Phoniatr Logop.

[123] Ziegler W, Steiger A. Motor speech impairment. In: Hickok G, Small SL, editors. Neurobiology of Language. 2016. pp. 985–94. 10.1016/B978-0-12-407794-2.00078-X.

[124] Müller J, Wenning GK, Verny M, McKee A, Chaudhuri KR, Jellinger K, Poewe W, Litvan I (2001). Progression of dysarthria and dysphagia in postmortem-confirmed parkinsonian disorders. Arch Neurol.

[125] Blumstein SE, Kurowski K (2006). The foreign accent syndrome: a perspective. J Neurolinguistics.

[126] Weintraub S, Mesulam MM, Kramer L (1981). Disturbances in prosody. A right-hemisphere contribution to language. Arch Neurol.

[127] Catsman-Berrevoets C, Paray Z. Cerebellar mutism syndrome. In: Manto M, Huisman TAGM, editors. The Cerebellum: Disorders and Treatment. Handbook of Clinical Neurology. 2018. pp. 2–424. 10.1016/B978-0-444-64189-2.00018-4. 10.1016/B978-0-444-64189-2.00018-4 29891065

[128] Ackermann H, Brendel B. Cerebellar contributions to speech and language Neurobiol Lang. 2016; Chapter 7. pp. 73–84 10.1016/B978-0-12-407794-2.00007-9

[129] Nicita F, Paiano M, Liberatore M, Spalice A, Papoff P, Ullo M (2017). Sudden benzodiazepine-induced resolution of post-operative pediatric cerebellar mutism syndrome: a clinical-SPECT study. Acta Neurochir.

[130] Lezak M, Howieson D, Bigler E, Tranel D (2012). Neuropsychological assessment.

[131] Shao Q, Guo Q, Hong Z (2013). Clustering and switching during a semantic verbal fluency contribute to differential diagnosis of cognitive impairment. Neurosci Bull.

[132] Bizzozero I, Scotti S, Clerici F, Pomati S, Laiacona M, Capitani E. On which abilities are category fluency and letter fluency grounded? A confirmatory factor analysis of 53 Alzheimer’s dementia patients. Dement Geriatr Cogn Dis Extra. 2013;3:179–91. 10.1159/000351418. Print 2013 Jan. 10.1159/000351418 PMC3711000 23885263

[133] Bellebaum C, Daum I (2007). Cerebellar involvement in executive control. Cerebellum..

[134] Leggio MG, Chiricozzi FR, Clausi S, Tedesco AM, Molinari M (2011). The neuropsychological profile of cerebellar damage: the sequencing hypothesis. Cortex..

[135] Raskin S, Sliwinski M, Borod JC (1992). Clustering strategies on tasks of verbal fluency in Parkinson’s disease. Neuropsychologia..

[136] Robbins TW, Cools R (2014). Cognitive deficits in Parkinson’s disease: a cognitive neuroscience perspective. Mov Disord.

[137] Petrides M, Pandya DN (2002). Comparative cytoarchitectonic analysis of the human and the macaque ventrolateral prefrontal cortex and corticocortical connection patterns in the monkey. Eur J Neurosci.

[138] Papagno C, Casarotti A, Comi A, Pisoni A, Lucchelli F, Bizzi A, Riva M, Bello L (2016). 2016. Long-term proper name anomia after removal of the uncinate fasciculus. Brain Struct Funct.

[139] Badre D, Wagner AD (2007). Left ventrolateral prefrontal cortex and the cognitive control of memory. Neuropsychologia..

[140] Thompson-Schill SL, D'Esposito M, Aguirre GK, Farah MJ (1997). Role of left inferior prefrontal cortex in retrieval of semantic knowledge: a reevaluation. Proc Natl Acad Sci U S A.

[141] Di Tella S, Baglio F, Pelizzari L (2020). Uncinate fasciculus and word selection processing in Parkinson’s disease. Neuropsychologia..

[142] Silveri MC, Traficante D, Lo Monaco MR, Iori L, Sarchioni F, Burani C (2018). Word selection processing in Parkinson’s disease: when nouns are more difficult than verbs. Cortex..

[143] Copland DA, Sefe G, Ashley J, Hudson C, Chenery HJ (2009). Impaired semantic inhibition during lexical ambiguity repetition in Parkinson’s disease. Cortex..

[144] Cappa SF (1997). Subcortical aphasia: still a useful concept?. Brain Lang.

[145] Nadeau SE, Crosson B (1997). Subcortical aphasia. Brain Lang.

[146] Miceli G, Denes G, Pizzamiglio L, Guariglia C, Cappa S, Grossi D, Luzzatti C (2019). Disturbi sintattici e dell’elaborazione di frasi nell’afasia. Manuale di neuropsicologia, normalità e patologia dei processi cognitivi.

[147] Ullman MT (2001). A neurocognitive perspective on language: the declarative/procedural model. Nat Rev Neurosci.

[148] Murdoch BE. The cerebellum and language: historical perspective and review. Cortex. 2010;46(7):858–68. 10.1016/j.cortex.2009.07.018. 10.1016/j.cortex.2009.07.018 19828143

[149] Bastiaanse R, Leenders KL (2009). Language and Parkinson’s disease. Cortex..

[150] Roussel M, Lhommée E, Narme P, Czernecki V, Gall DL, Krystkowiak P, Diouf M, Godefroy O, on behalf of the GREFEX study group, GREFEX study group (2017). Dysexecutive syndrome in Parkinson’s disease: the GREFEX study. Aging Neuropsychol Cogn.

[151] Crosson B (2013). Thalamic mechanisms in language: a reconsideration based on recent findings and concepts. Brain Lang.

[152] Schmahmann JD (2003). Vascular syndromes of the thalamus. Stroke..

[153] Barbas H, García-Cabezas MÁ, Zikopoulos B (2013). Frontal-thalamic circuits associated with language. Brain Lang.

[154] Wyman-Chick KA (2016). Verbal fluency in Parkinson’s patients with and without bilateral deep brain stimulation of the subthalamic nucleus: a meta-analysis. J Int Neuropsychol Soc.

[155] Xu W, Russo GS, Hashimoto T, Zhang J, Vitek JL (2008). Subthalamic nucleus stimulation modulates thalamic neuronal activity. J Neurosci.

[156] Boes AD, Prasad S, Liu H, Liu Q, Pascual-Leone A, Caviness VS, Fox MD (2015). Network localization of neurological symptoms from focal brain lesions. Brain..

[157] Brettschneider J, Suh E, Robinson JL, Fang L, Lee EB, Irwin DJ, Grossman M, van Deerlin VM, Lee VMY, Trojanowski JQ (2018). Converging patterns of α-synuclein pathology in multiple system atrophy. J Neuropathol Exp Neurol.

[158] Leggio M, Olivito G, Lupo M, Clausi S. The cerebellum: A therapeutic target in treating speech and language disorders. In: Argyropoulos G, editor. Translational Neuroscience of Speech and Language Disorders. Contemporary Clinical Neuroscience. Springer, Cham. 2020. pp. 141–75. 10.1007/978-3-030-35687-3_8.

